# Uncertainty maps for model-based global climate classification systems

**DOI:** 10.1038/s41597-025-04387-0

**Published:** 2025-01-08

**Authors:** Andrés Navarro, Andrés Merino, Eduardo García-Ortega, Francisco J. Tapiador

**Affiliations:** 1 Earth and Space Sciences (ESS) Group, Institute of Environmental Sciences, University of Castilla-La Mancha (UCLM), Avda. Carlos III s/n, 45071 Toledo, Spain; 2 Atmospheric Physics Group (GFA), Environmental Institute, Universidad de León (ULE), Calle de la Serna 58, 24007 León, Spain

**Keywords:** Climate change, Environmental sciences

## Abstract

Climate classification systems (CCSs) are emerging as essential tools in climate change science for mitigation and adaptation. However, their limitations are often misunderstood by non-specialists. This situation is especially acute when the CCSs are derived from Global Climate Model outputs (GCMs). We present a set of uncertainty maps of four widely used schemes -Whittaker-Ricklefs, Holdridge, Thornthwaite-Feddema and Köppen- for present (1980–2014) and future (2015–2100) climate based on 52 models from the Coupled Intercomparison Model Project Phase six (CMIP6). Together with the classification maps, the uncertainty maps provide essential guidance on where the models perform within limits, and where sources of error lie. We share a digital resource that can be readily and freely integrated into mitigation and adaptation studies and which is helpful for scientists and practitioners using climate classifications, minimizing the risk of pitfalls or unsubstantiated conclusions.

## Background & Summary

Climate classification systems (CCSs) are valuable tools for societal and environmental applications^1–3^. They simplify complex, multidimensional climate data by transforming continuous variables, such as temperature and precipitation, into discrete categories that are meaningful for ecological purposes. This process, known as dimension reduction, creates a user-friendly format that facilitates the identification of broad patterns between climate drivers and the spatial distribution of biota. Used together with climate model outputs, CCSs provide a neat description of the past and future climate change^4–7^.

A significant limitation of CCSs, however, is that their ability to accurately define climate zones depends on the quality of the input data. If these are uncertain, the resulting product is compromised. End users assume these uncertainties are monitored by the developers of CCSs and that their impact on the final product is limited, giving a false sense of confidence^8^.

Most CCSs are built using monthly and/or yearly data from two primary variables: temperature (TS) and precipitation (PR). TS is a smooth, highly spatially autocorrelated climate field. In contrast, PR exhibits high spatial variability, making it challenging to measure and predict. Indeed, errors exceeding 100% are not uncommon for precipitation data^9^. Given the many difficulties in capturing the true nature of precipitation, it has unsurprisingly become the primary source of uncertainty and bias in CCSs, potentially limiting the reliability of the analysis derived from these datasets^10^.

**Fig. 1:**
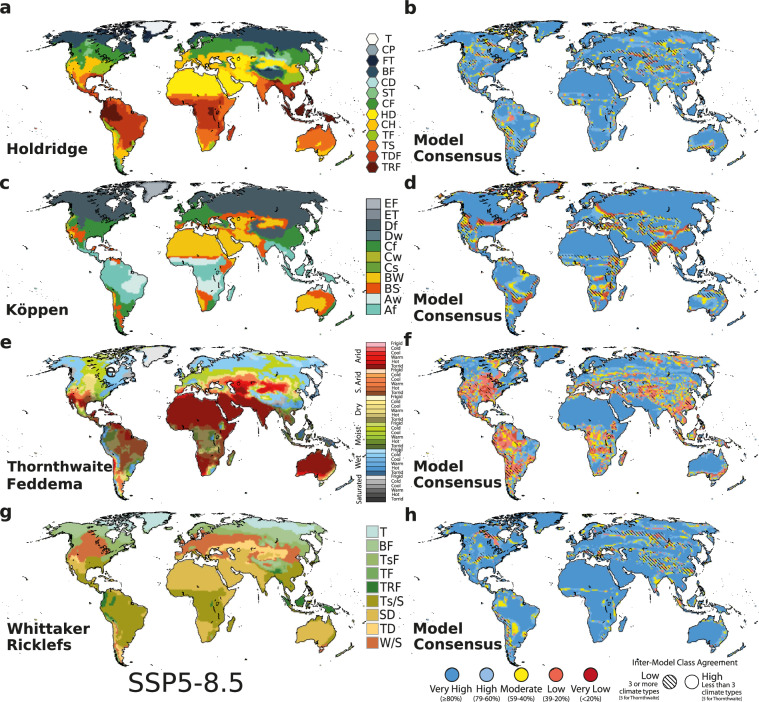
Global distribution of climate zones for the four CCSs for the SSP5-8.5 future scenario (**a,c,e,g**). Consensus maps of climate zones for the top-10 ensemble mean (**b,d,f,h**). Colors represent the degree of confidence: dark blue (≥80%), light blue (79-60%), yellow (59-40%), light red (39-20%), and dark red (<20%). The stripes show the regions with low class agreement.

Our goal is to provide both practitioners and researchers from a variety of disciplines -e.g., biologists, geographers, and ecologists- with the tools and datasets they need for accurate environmental analysis based on the current knowledge of state-of-the-art Global Climate Models (GCMs). A key element is that of *consensus maps*, as defined by Navarro *et al*.^10^. These maps, along with their corresponding classification maps, synthesize information from multiple models to reveal areas of agreement (or disagreement) according to present and future climate types (Fig. 1). This comprehensive view allows users to navigate potential pitfalls in their analyses and ensure robust research.

This analysis leverages output from top-ranked GCMs to explore future climate conditions through the lens of four well-known climate classification systems: Whittaker-Ricklefs biomes, Köppen’s climate types, Thornthwaite-Feddema climate classification, and Holdridge’s life zones. While Köppen is dominant^11–13^, the other CCSs offer valuable and often more nuanced perspectives on the complex relationship between climate and environment, each tailored to the needs of specific audiences^14–16^.

## Methods

### Climate Classification Systems

The main four CCSs used in the literature are included: 1. Holdridge’s life zones^17^; 2. Köppen climate types^18^; 3. Thornthwaite’s classification^19^; 4. Whittaker’s biomes^20^.

Holdridge life zones are defined by three measurements: annual precipitation (mm·year^−1^), biotemperature (°C) and potential evapotranspiration (PET) ratio. Annual precipitation is calculated from monthly precipitation data. Mean annual biotemperature is derived from monthly average temperature. Those months with a mean temperature above 30.0 °C and below 0.0 °C are considered as 30.0 °C and 0.0 °C, respectively. PET ratio is defined as the mean annual biotemperature multiplied by a constant value (58.93) and divided by annual precipitation. We assign a class to each grid cell by computing the minimum Euclidean distance between each pixel and the geometric centroids of life zones, as defined in Sisneros *et al*.^21^. The 33 classes are then grouped into 13 categories as shown in Table 1.

**Table 1:** Holdridge life zones and their geometric centroids, as distributed in 13 major biomes.

		Geometric centroids		
Biome	Holdridge Life Zone	Precipitation (mm yr^−1^)	Biotemperature (°C)	PET ratio
Tundra (T)	Polar Desert	88.39	0.00	0.71
	Polar Desert	176.78	0.00	0.35
	Polar Desert	353.55	0.00	0.18
Cold Parklands (CP)	Dry Tundra	88.39	2.12	1.41
	Boreal Desert	88.39	4.24	2.83
	Dry Scrub	176.78	4.24	1.41
Forest Tundra (FT)	Moist Tundra	176.78	2.12	0.71
	Wet Tundra	353.55	2.12	0.35
	Rain Tundra	707.11	2.12	0.18
Boreal Forest (BF)	Moist Forest	353.55	4.24	0.71
	Wet Forest	707.11	4.24	0.35
	Rain Forest	1414.21	4.24	0.18
Cool Desert (CD)	Montane Desert	88.39	8.49	5.66
	Desert Scrub	176.78	8.49	2.83
Steppe (ST)	Steppe	353.55	8.49	1.41
Cool Forest (CF)	Moist Forest	707.11	8.49	0.71
	Wet Forest	1414.21	8.49	0.35
	Rain Forest	2828.43	8.49	0.18
Hot Desert (HD)	Subtropical Desert	88.39	16.97	11.31
	Desert Scrub	176.78	16.97	5.66
	Tropical Desert	88.39	26.83	22.63
	Desert Scrub	176.78	26.83	11.31
Chaparral (CH)	Thorn Steppe/Woodland	353.55	16.97	2.83
	Dry Forest	707.11	16.97	1.41
Temperate Forest (TF)	Moist Forest	1414.21	16.97	0.71
	Wet Forest	2828.43	16.97	0.35
	Rain Forest	5656.85	16.97	0.18
Tropical Semi-arid (TS)	Thorn Woodland	353.55	26.83	5.66
	Very Dry Forest	707.11	26.83	2.83
Tropical Dry Forest (TDR)	Dry Forest	1414.21	26.83	1.41
Tropical Rain Forest (TRF)	Moist Forest	2828.43	26.83	0.71
	Wet Forest	5656.85	26.83	0.35
	Rain Forest	11313.71	26.83	0.18

Adapted from Sisneros *et al*.^21^ and Monserud and Leemans^42^.

For the Köppen scheme, we followed the work of Lohman *et al*.^22^, which is based on Köppen’s early work. In this seminal publication, ecoregions are divided into five climate types, four thermal types (A, C, D, E), and one hydrologic type (B). In addition, the scheme includes three subtypes (f, s, w) related to the annual cycle of precipitation. Operationally, the classification algorithm works as follows: first, it evaluates the precipitation threshold. Second, it processes climate types A, C, D and E, respectively. This workflow ensures a consistent identification of arid climates^23^. Otherwise, discrepancies may arise. While this is not a problem when only models are compared (as in Tapiador *et al*.^24^), it will certainly yield paradoxes if observations are used. The defining criteria of each climate type are shown in Table 2.

**Table 2:** Climate types and defining criteria for Köppen.

Class	Type	Criteria
**A** (Tropical)		T_min ≥ 18 °C
	**Af**. Tropical rainforest	P_min ≥ 60 mm
	**Aw**. Tropical savanna	P_min < 60 mm
**B** (Dry)		P_annual ≤ P_threshold
	**BS**. Steppe	P_annual ≥ P_threshold/2
	**BW**. Desert	P_annual < P_threshold/2
**C** (Mesothermal)		T_min ≥ −3 °C & T_min < 18 °C
	**Cs**. Warm climate dry summer	P_wmax ≥ 3 × P_smin
	**Cw**. Warm climate dry winter	P_smax ≥ 10 × P_wmin
	**Cf**. Humid temperate	P_smax < 10 × P_wmin & P_wmax < 3 × P_smin
**D** (Microthermal)		T_min < −3 °C & T_max > 10 °C
	**Dw**. Cold climate dry winter	P_smax ≥ 10 × P_wmin
	**Df**. Cold climate moist winter	P_smax < 10 × P_wmin
**E** (Polar)		T_max < 10 °C
	**ET**. Tundra	T_max ≥ 0 °C & T_max < 10 °C
	**EF**. Permafrost	T_max < 0 °C

T_min = temperature coldest month, T_max = temperature hottest month, P_min = precipitation driest month, P_annual = annual precipitation, P_smin = minimum summer precipitation, P_smax = maximum summer precipitation, P_wmin = minimum winter precipitation, P_wmax = maximum winter precipitation, P_threshold = varies according to the following rules (if 70% of P_annual occurs in winter then P_threshold = 2 × T_avg; if 70% of P_annual occurs in summer then P_threshold = 2 × T_avg + 28; otherwise P_threshold = 2 × T_avg + 14).

We used the revised version of Thornthwaite’s classification proposed by Feddema^25^, which has become standard practice in the field. Similar to the original work, it centers on the concept of water availability. However, this revision incorporates a simplified version of the moisture state, as defined by Willmott and Feddema^26^. Moisture Index *I*_*m*_ ranges from −1 to 1 and is defined as follows: $${I}_{m}=\left\{\begin{array}{c}\left(r/{PE}\right)-1,r < {PE}\\ 1-\left({PE}/r\right),r\ge {PE}\end{array}\right.$$ where *r* is the annual rainfall and *PE* is potential evapotranspiration. The thermal component remains faithful to the classic definition of potential evapotranspiration developed by Thornthwaite. Table 3 shows the criteria defining the 36 climate types.

**Table 3:** Description of thermal and moisture types according to Feddema’s method.

Thermal Type	Annual Potential Evapotranspiration [mm]	Moisture Type	Moisture Index
Torrid	>1500	Saturated	0.66–1.0
Hot	1200–1500	Wet	0.33–0.66
Warm	900–1200	Moist	0.0–0.33
Cool	600–900	Dry	−0.33–0.0
Cold	300–600	Semiarid	−0.66 – −0.33
Frigid	0–300	Arid	−1.0 − −0.66

**Fig. 2:**
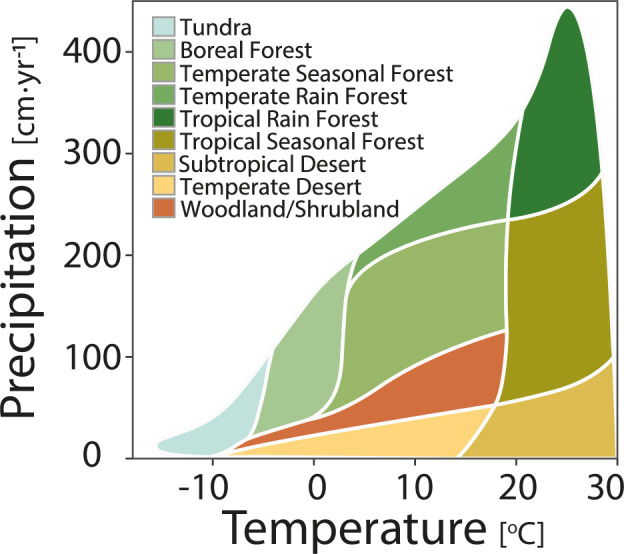
Whittaker’s Biome Diagram. Whittaker’s scheme uses climatologies of precipitation and temperature to shape the spatial pattern of major biomes. This is a modified version of Whittaker’s original work based on Ricklefs’s diagram.

We followed the delineation of Whittaker’s biomes as described by Ricklefs^27^. The scheme divides the Earth into nine biomes according to annual precipitation (cm·year^−1^) and annual mean temperature (^o^C). The biome distribution criteria are shown in Fig. 2.

Classification for this case was performed using the R package *plotbiomes*^28^. Outliers were assigned the climate type of the closest biome.

### Climate data

Climate classification maps and consensus maps were constructed using monthly precipitation and temperature from 52 models of the Coupled Model Intercomparison Project phase 6 (CMIP6)^29^. CMIP6 data are publicly available and have been downloaded from the Earth System Grid Federation node (https://aims2.llnl.gov/search). The dataset included both the current climate (1980–2014) and future projections (2021–2050, 2051–2100, 2015–2100). To explore a range of future scenarios, we incorporated data from three Shared Socioeconomic Pathways (SSPs)^30^: SSP1-2.6, SSP2-4.5, and SSP5-8.5. The three choices are intended to be comprehensive and cover the vast majority of needs of adaptation and mitigation studies. Finally, to ensure consistency, all datasets were interpolated to a common horizontal resolution of 1° × 1°, using bilinear interpolation. This choice aligns with the native resolution of most CMIP6 models and minimizes potential uncertainties introduced by downscaling to finer resolutions, which is critical for developing consensus maps. The analysis was for land-only, excluding Antarctica. The full list of models can be found in Supplementary Information (Table S1).

### Model rank and top-10 ensemble members

We used Cohen’s kappa coefficient^31^ to quantify the ability of each model to reproduce the distribution of climate categories. This standard method is widely used to gauge model quality. Present reference climate is also the standard choice: it is calculated using the Climate Research Unit Time Series (CRU, version 4.04^32^) observations for each CCS for the historical climatological period (1980–2014). The kappa coefficient (κ) is defined as follows: ${\rm{\kappa }}=\frac{{P}_{0}-{P}_{e}}{1-{P}_{e}}$, where *P*_0_ is the proportion of units with agreement and *P*_*e*_ is the hypothetical probability of chance agreement. The kappa statistic ranges from 0 to 1, with 0 meaning no agreement and 1 meaning perfect agreement.

**Fig. 3:**
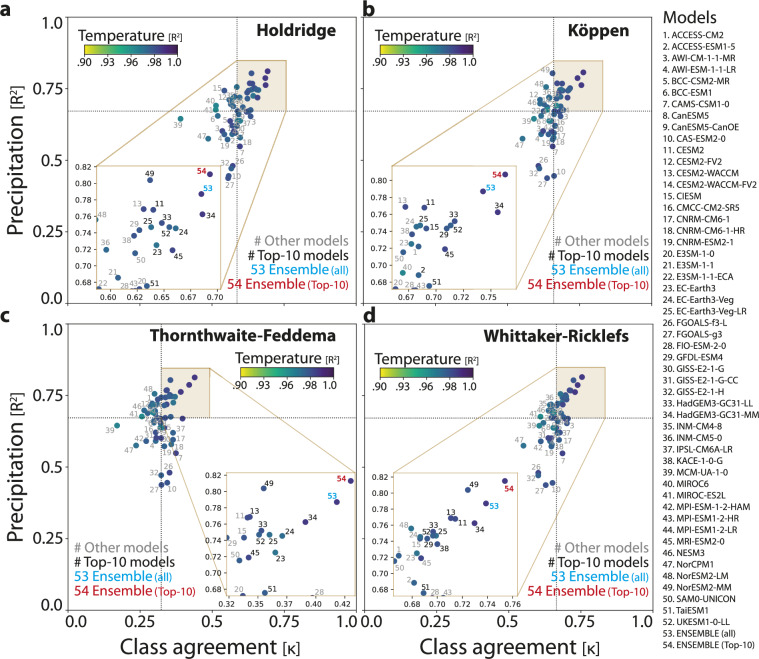
Scores of CMIP6 models for the four classification schemes: (**a**) Holdridge, (**b**) Köppen, (**c**) Thornthwaite-Feddema, (**d**) Whittaker-Ricklefs. Dots are individual CMIP6 models (52 models + 2 ensemble means). The *y axis* is the R^2^ score for global precipitation (mm·year^−1^), *x axis* is the kappa coefficient of each CCS and colors are the R^2^ score for global mean temperature. Dotted grey lines are the median. Zoomed areas indicate the region of best performance, and models within this area are then selected to create the top-10 ensemble mean (T10). Numbers in black are the top 10 models, while the other models are in grey. The red number is the T10 ensemble mean, and the blue one is the 52-model ensemble mean. The analysis was performed for the 1980–2014 period.

Models were also ranked in terms of joint agreement with precipitation and temperature observations. The metric used for these quantitative variables was the coefficient of determination *R*^2^. The reason for using this metric is twofold: first, it has been shown to be useful for comparing large-scale means of climate data, such as those used by most climate classification schemes^15^. Second, it condenses information into a single, easy-to-interpret value, thus facilitating comparisons between models. Grid boxes were weighted by area in both metrics. We then identified the best performing models by focusing on the upper right quadrant of a 2D plane (Fig. 3). This quadrant represents models with both high class agreement (κ) and high precipitation scores (R²). The median values of κ and R² (precipitation) were used to define the boundaries. Finally, we selected the top-10 models within this quadrant based on the highest κ scores.

Table 4 shows the scores for the top-10 models for Whittaker’s scheme. For the remaining CCSs, see Tables S2–S4 in the Supplementary Information document.

**Table 4:** Scores of top-10 models, (52) ensemble, and (T10) ensemble for Whittaker’s biomes.

Model	Climate Cats. (ĸ)	Precipitation (R^2^)	Temperature (R^2^)
CESM2	0.714	0.768	0.977
CESM2-WACCM	0.711	0.769	0.978
EC-Earth3-Veg-LR	0.699	0.747	0.964
GFDL-ESM4	0.692	0.743	0.973
HadGEM3-GC31-LL	0.697	0.752	0.976
HadGEM3-GC31-MM	0.730	0.762	0.983
KACE-1-0-G	0.700	0.736	0.974
NorESM2-MM	0.724	0.804	0.973
TaiESM1	0.689	0.676	0.971
UKESM1-0-LL	0.697	0.747	0.970
Ensemble (52 models)	0.739	0.787	0.983
Top-10 ensemble	0.759	0.815	0.983

### Uncertainty analysis

**Fig. 4:**
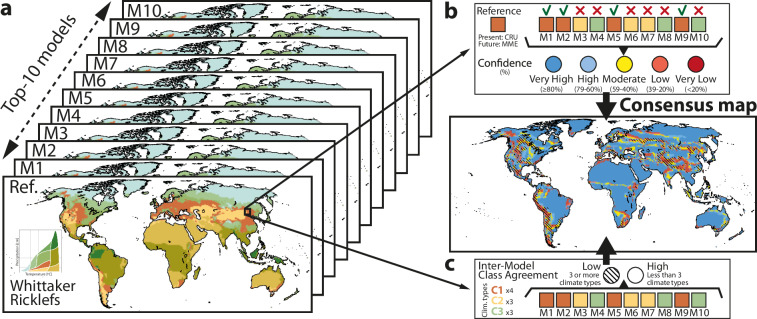
Graphical description of the uncertainty estimation of distributed climate zones. (**a**) Maps of climate types for top-10 models and reference data. (**b**) The performance of individual models was evaluated against the reference data in a pixel-by-pixel comparison, and the degree of agreement between reference and models (in percent) was then calculated. Each pixel is colorized in the consensus map according to the level of confidence: dark blue (≥80%), light blue (79-60%), yellow (59-40%), light red (39-20%), and dark red (<20%). (**c**) Inter-model class agreement, which represents the number of different climate types identified by the top-10 models for each pixel. Two or fewer climate types indicates low inter-model variability, while three or more indicates high variability (striped regions in the consensus map). The analysis was performed for the four climate classification schemes. Reference data is CRU (for present) and T10 ensemble mean (for future scenarios).

Model uncertainties are embedded in the so-called *consensus maps* (Fig. 4). Consensus maps provide a qualitative description of model uncertainties, highlighting regions where models disagree. Quantitatively, these maps are built on two key metrics derived from pixel-by-pixel comparisons: percent confidence and inter-model class agreement.

The first metric (Fig. 4b) quantifies the accuracy of individual models relative to observed climate types. The formulation is as follows: ${{\rm{Confidence}}}_{i,j}=\frac{{{coincident\; models}}_{i,j}}{{{Nmodels}}_{i,j}}\cdot 100$, where the fraction represents the proportion of models, for each pixel (i,j), that predicted a climate class matching the reference data. Here, coincident models refers to the number of models with matching climate type, and *Nmodels* represents the total number of models considered. Confidence levels were discretized into five categories: very high (≥80%), high (79%- 60%) moderate (59%-40%), low (39%-20%), and very low (<20%). The second metric evaluates inter-model variability, i.e. how many climate types are identified by different models for the same point in space. For example, Fig. 4c shows that for a given pixel at (i,j), four models defined the region as woodland, three as temperate desert, and the other three as boreal forest. This results in three different climate types, which implies low inter-model agreement. Both metrics are complementary, the first metric shows the accuracy while the second measures the dispersion (precision). Uncertainty maps were constructed for each CCS for the present and the three SSPs, using the CRU and the top-10 ensemble, respectively, as reference.

## Data Records

Global datasets of the four CCSs and their associated consensus maps for present and future climates are available at Figshare^33^. The data are provided in three different file formats to meet the needs of a wide range of users. All datasets have a spatial resolution of 1°, which corresponds to 180 × 360 pixels. For ease of download, individual files within the same format are combined into a single tar.gz archive.

1. GeoTIFF files are for general users who want an immediate image of the distribution of climate types and consensus maps. The compressed file contains 120 GeoTIFF files accompanied by a legend (legend.txt) that defines the code categories used in each climate classification system (13 for Holdridge, 11 for Köppen, 36 for revised Thornthwaite and 9 for Whittaker’s biomes). The filename structure is: classification_varname_scenario_period.tif (e.g *koppen_confidence_historical_1980-2014.tif*, *whittaker_class_ssp126_2051-2100.tif*, and *koppen_modvar_ssp585_2021-2050.tif*). *Class* refers to climate classification, *confidence* is percent confidence, and *modvar* refers to inter-model variability.
2. NetCDF files are for more advanced users. They can be directly read in a Geographic Information System (GIS) such as QGIS. A major advantage is that they are self-describing files, meaning the data and its metadata are stored together in a single file. The tar.gz includes 80 nc files. The files including climate classification maps also include their respective key variables, except for Köppen. For example, the file *whittaker_class_historical_1980-2014.nc* contains data for annual mean temperature (*AT*, in ^o^C), annual precipitation (*APP*, in cm·year^-1^), and Whittaker’s climate classification itself. Similarly, *holdridge_class_ssp245_2051*–*2100.nc* includes biotemperature (*ABT*, in ^o^C), annual precipitation (*APP*, in mm·year^−1^), potential evapotranspiration ratio (PER) and Holdridge’s life zones (HLZ) for the future scenario SSP2-4.5 and the period 2051-2100. Files of consensus maps, like *holdridge_consensus_ssp585_2021-2050.nc*, store the following variables: *confidence* and *modvar*. Codes for the classification categories are embedded within the file’s attributes for easy reference.
3. BIL, pure binary files are suited for fast reading and efficient computation. The compressed file contains 120 bil files, along with the corresponding header files (hdr) and a legend file (legend.txt). They can also be directly read in a GIS. The dimensions of each bil file are 180 × 360. Filenames follow the same naming convention as tiff files.

The data are based on the top-10 ensemble mean, although we performed the calculations for the 52 CMIP6 models. Data for individual models and the MME (52) are available upon request to the corresponding author.

## Technical Validation

### Model validation

Climate classifications derived from GCMs were validated against observations from the Climate Research Unit (CRU at the University of East Anglia) Time Series version 4.04^32^. The CRU dataset has 0.5° spatial resolution but was aggregated to 1°, following the same procedure as for the CMIP6 models. The validation was performed for three variables: annual precipitation, mean annual temperature, and climate classes. Figure 3 shows the scores of individual models, as well as ensemble means -MME (52) and top-10 ensemble- for the 1980–2014 period. Alternative ensemble sizes (top-20, top-30 and top-40) were also evaluated, but their performance did not show consistent improvement over top-10 and MME. As illustrated in the figure, global performance varies depending on the climate classification scheme. Whittaker’s biomes achieve the highest overall scores (κ = 0.74), while Thornthwaite’s shows the lowest scores (κ = 0.42) for the MME (52). Eight individual GCMs/ESMs consistently achieve the highest scores across all CCSs: CESM2, EC-Earth-Veg-LR, HadGEM3-GC31-LL, HadGEM3-GC31-MM, MRI-ESM2-0, NorESM2-MM, TaiESM1 and UKESM1-0-LL. This is likely because they perform better at simulating precipitation, a key source of bias in CCSs^10^. For mean temperature, all models obtained scores above R^2^ = 0.95.

### Uncertainty analysis of reference data

Observational data often have differences that can affect the representation of climate zones. To assess this sensitivity, we evaluated climate classifications using alternative datasets in addition to CRU. These included station-based data from the University of Delaware (UDEL version 5.01^34^) and the Global Precipitation Climatology Centre (GPCC) Full Data Monthly Product version 2022^35,36^, as well as the ECMWF’s reanalysis data^37,38^ (ERA5).

**Fig. 5:**
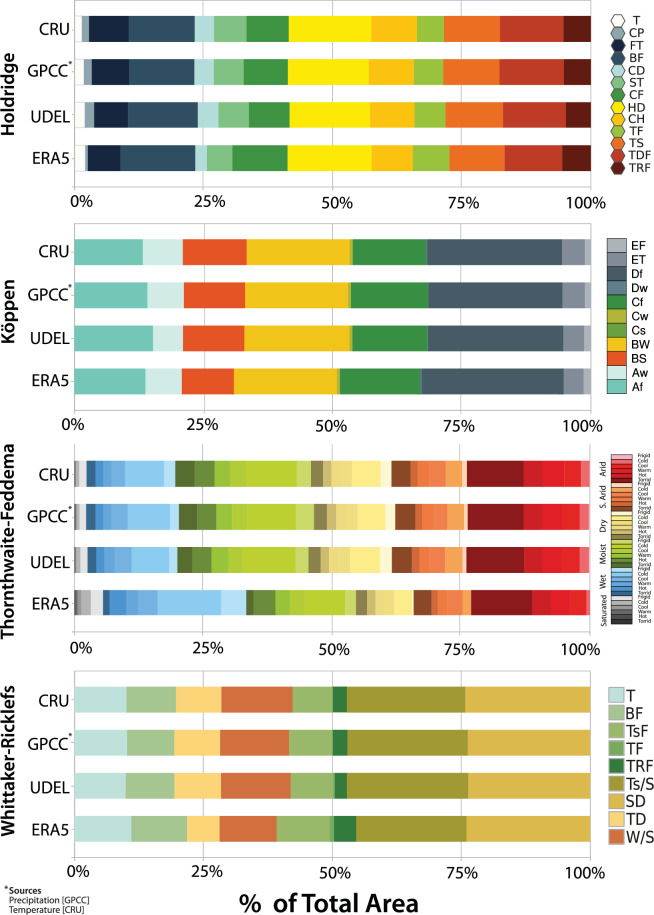
Percentage of continental area covered by each climate type for the four climate classification schemes (Köppen, Holdridge, Thornthwaite-Feddema, and Whittaker-Ricklefs) and the four observational datasets (CRU, GPCC, UDEL and ERA5). Classification schemes for GPCC are a combination of data from the GPCC (precipitation) and CRU (temperature).

A classification scheme that is insensitive to these variations is considered robust to observational uncertainties^39^. Figure 5 illustrates this concept, showing small changes in the global distribution of climate types.

CRU and GPCC show the greatest agreement (1.3% to 1.6% of the discrepancy in total land area) for Holdridge, Köppen, and Whittaker classifications. UDEL also has relatively small discrepancies (1.5% to 3.1%), while ERA5 displays slightly larger differences (3.6% to 7.3%). Thornthwaite’s scheme shows the greatest sensitivity to data source. Here, ERA5 predicts a shift in high-latitude climates, with cold-wet types becoming more common at the expense of cold-moist types.

## Usage Notes

The dataset offers a comprehensive description of current and future global climate, based on four major classification schemes. We provide maps for Holdridge, Köppen, Thornthwaite-Feddema, and Whittaker-Ricklefs climate classifications along with their corresponding consensus maps. Future scenarios included are SSP1-2.6, SSP2-4.5 and SSP5-8.5 (three time-periods: 2021-2050, 2051-2100, and 2015-2100). The data are derived from an ensemble of the top-10 CMIP6 models (out of 52), selected based on their kappa statistic and their R^2^ score for precipitation. Top-10 models differ by classification scheme. Validation with observations (CRU) indicates that model outputs are reasonable. However, there are certain caveats that users may consider before using the dataset:

1. We assume that current criteria for defining climate types are the same for future climate, leaving no room for new equilibrium conditions or new climate types. This is a common issue in any rule-based classification scheme^40,41^.
2. Our maps have a 1° spatial resolution, which is the native resolution of most CMIP6 models. While it might be deemed low for certain regional applications, it is worth noting that kilometer or even hectometer resolutions are the result of interpolations well below the resolution of the original empirical data. Indeed, there is no limit for downscaling a scalar field; the only question is what is sacrificed in the mathematical process. By keeping the spatial resolution at the nominal model grid size, we avoid introducing additional uncertainties associated with downscaling techniques, and ensure that the final product reflects the inherent resolution of the underlying climate models.
3. Model performance is contingent to the classification scheme. As shown in Fig. 3, individual models and ensemble means presented different scores, according to the CCS. While models performed well for Köppen (11 cats), Holdridge (13 cats), and Whittaker (9 cats), the performance for Thornthwaite (36 cats) was moderate, meaning that their maps should be used with caution.
4. All four CCSs are primarily based on precipitation and temperature. However, each CCS was developed with distinct goals and criteria, leading to variations in how they identify regions undergoing climate change.
5. Consensus maps depict the confidence of model predictions pinpointing regions where consensus is lacking. While many of such regions overlap across CCSs, some disagreements arise, specifically at the boundary of climate types, as defined by each unique classification scheme. Additionally, the degree of consensus may vary depending on the socio-economic scenario chosen.

## Supplementary information

Supplementary Tables

## Data Availability

The codes for the CCSs and consensus maps are available on GitHub at https://github.com/navarro-esm/uncertainty_maps_library. The source code of Whittaker’s biomes is available from https://github.com/valentinitnelav/plotbiomes.
