# Supplementary material for: Uncertainty maps for model-based global climate classification systems
Source: Sci Data. 2025 Jan 8;12:35. doi: 10.1038/s41597-025-04387-0 (PMC11711615; doi:10.1038/s41597-025-04387-0)
Supplement: Supplementary file 1 — Supplementary Tables [file 41597_2025_4387_MOESM1_ESM.docx]

**Supplementary Table S1.** List of CMIP6 models and reference dataset used in the present study.

| **Model Name** | **Horizontal resolution** (lat x lon) | **Variant label** | **Institution / Country** | **Reference** |
| --- | --- | --- | --- | --- |
| ACCESS-CM2 | 1.3^o^ × 1.9^o^ | r1i1p1f1 | Commonwealth Scientific and Industrial Research Organisation; Australian Research Council Centre of Excellence for Climate System Science (CSIRO-ARCCSS) /Australia | Bi et al 2020 |
| ACCESS-ESM1-5 | 1.2^o^ × 1.9^o^ | r1i1p1f1 | Commonwealth Scientific and Industrial Research Organisation (CSIRO) / Australia | Ziehn et al 2020 |
| AWI-CM-1-1-MR | 0.9^o^ × 0.9^o^ | r1i1p1f1 | Alfred Wegner Institute (AWI) / Germany | Semmler et al. 2020 |
| AWI-ESM-1-1-LR | 1.9^o^ × 1.9^o^ | r1i1p1f1 |  |  |
| BCC-CSM2-MR | 1.1^o^ × 1.1^o^ | r1i1p1f1 | Beijing Climate Center (BCC) /China | Wu et al 2019 |
| BCC-ESM1 | 2.8^o^ × 2.8^o^ | r1i1p1f1 |  | Wu et al 2020 |
| CAMS-CSM1-0 | 1.1^o^ × 1.1^o^ | r1i1p1f1 | Chinese Academy of Meteorological Sciences (CAMS) / China | Rong et al 2019 |
| CanESM5 | 2.8^o^ × 2.8^o^ | r1i1p1f1 | Canadian Centre for Climate Modelling and Analysis, Environment and Climate Change (CCCma) / Canada | Swart et al 2019 |
| CanESM5-CanOE | 2.8^o^ × 2.8^o^ | r1i1p2f1 |  |  |
| CAS-ESM2-0 | 1.4^o^ × 1.4^o^ | r1i1p1f1 | Chinese Academy of Sciences (CAS) / China | Zhou et al 2020 |
| CESM2 | 0.9^o^ × 1.3^o^ | r1i1p1f1 | National Center for Atmospheric Research, Climate and Global Dynamics Laboratory (NCAR) / USA | Danabasoglu et al 2020 |
| CESM2-FV2 | 1.9^o^ × 2.5^o^ | r1i1p1f1 |  |  |
| CESM2-WACCM | 0.9^o^ × 1.3^o^ | r1i1p1f1 |  |  |
| CESM2-WACCM-FV2 | 1.9^o^ × 2.5^o^ | r1i1p1f1 |  |  |
| CIESM | 0.9^o^ × 1.3^o^ | r1i1p1f1 | Tsinghua University. Department of Earth System Science. (TSU) / China | Lin et al 2020 |
| CMCC-CM2-SR5 | 0.9^o^ × 1.3^o^ | r1i1p1f1 | Fondazione Centro Euro-Mediterraneo sui Cambiamenti Climatici (CMCC) / Italy | Cherchi et al 2019 |
| CNRM-CM6-1 | 1.4^o^ × 1.4^o^ | r1i1p1f2 | Centre National de Recherches Meteorologiques; Centre Européen de Recherche et de Formation Avancée en Calcul Scientifique (CNRM-CERFACS) / France | Voldoire et al 2019 |
| CNRM-CM6-1-HR | 0.5^o^ × 0.5^o^ | r1i1p1f2 |  |  |
| CNRM-ESM2-1 | 2.8^o^ × 1.4^o^ | r1i1p1f2 |  | Séférian et al 2019 |
| E3SM-1-0 | 1.0^o^ × 1.0^o^ | r1i1p1f1 | E3SM-Project.  Lawrence Livermore National Laboratory (LLNL); Argonne National Laboratory, Argonne (ANL); Brookhaven National Laboratory (BNL); Los Alamos National Laboratory (LANL); Lawrence Berkeley National Laboratory (LBNL); Oak Ridge National Laboratory (ORNL); Pacific Northwest National Laboratory (PNNL); Sandia National Laboratories (SNL) / USA | Golaz et al 2019 |
| E3SM-1-1 | 1.0^o^ × 1.0^o^ | r1i1p1f1 |  |  |
| E3SM-1-1-ECA | 1.0^o^ × 1.0^o^ | r1i1p1f1 |  |  |
| EC-Earth3 | 0.7^o^ × 0.7^o^ | r1i1p1f1 | EC-Earth Consortium / Europe | Döscher et al 2022; Wyser et al 2020 |
| EC-Earth3-Veg | 0.7^o^ × 0.7^o^ | r1i1p1f1 |  |  |
| EC-Earth3-Veg-LR | 1.1^o^ × 1.1^o^ | r1i1p1f1 |  |  |
| FGOALS-f3-L | 1.0^o^ × 1.3^o^ | r1i1p1f1 | Chinese Academy of Sciences (CAS) / China | He et al 2019 |
| FGOALS-g3 | 2.3^o^ × 1.3^o^ | r1i1p1f1 |  | Li et al 2020 |
| FIO-ESM-2-0 | 0.9^o^ × 1.3^o^ | r1i1p1f1 | First Institute of Oceanography; Qingdao National Laboratory for Marine Science and Technology (FIO-QNLM) / China | Bao et al 2020 |
| GFDL-ESM4 | 0.9^o^ × 1.3^o^ | r1i1p1f1 | National Oceanic and Atmospheric Administration; Geophysical Fluid Dynamics Laboratory (NOAA-GFDL) / USA | Dunne et al 2020 |
| GISS-E2-1-G | 2.0^o^ × 2.5^o^ | r1i1p1f1 | Goddard Institute for Space Studies (NASA-GISS) / USA | Kelley et al 2020 |
| GISS-E2-1-CC | 2.0^o^ × 2.5^o^ | r1i1p1f1 |  |  |
| GISS-E2-1-H | 2.0^o^ × 2.5^o^ | r1i1p1f1 |  |  |
| HadGEM3-GC31-LL | 1.3^o^ × 1.9^o^ | r1i1p1f3 | Met Office Hadley Centre (MOHC) / UK | Kuhlbrodt et al 2018 |
| HadGEM3-GC31-MM | 0.6^o^ × 0.8^o^ | r1i1p1f3 |  | Andrews et al 2020 |
| INM-CM4-8 | 1.5^o^ × 2.0^o^ | r1i1p1f1 | Institute for Numerical Mathematics (INM) / Russian Federation | Volodin et al 2018 |
| INM-CM5-0 | 1.5^o^ × 2.0^o^ | r1i1p1f1 |  | Volodin and Gritsun 2018 |
| IPSL-CM6A-LR | 1.3^o^ × 2.5^o^ | r1i1p1f1 | Institut Pierre Simon Laplace (IPSL) / France | Boucher et al 2020 |
| KACE-1-0-G | 1.3^o^ × 1.9^o^ | r1i1p1f1 | National Institute of Meteorological Sciences/Korea Meteorological Administration (NIMS-KMA) / Rep. of Korea | J. Lee et al 2020 |
| MCM-UA-1-0 | 2.3^o^ × 3.8^o^ | r1i1p1f1 | Department of Geosciences, University of Arizona (UA) / USA | Delworth et al 2002 |
| MIROC6 | 2.8^o^ × 1.4^o^ | r1i1p1f1 | Japan Agency for Marine-Earth Science and Technology (JAMSTEC); Atmosphere and Ocean Research Institute (AORI); National Institute for Environmental Studies (NIES); RIKEN Center for Computational Science (R-CCS) / Japan | Tatebe et al 2019 |
| MIROC-ES2L | 2.8^o^ × 2.8^o^ | r1i1p1f2 |  | Hajima et al 2020 |
| MPI-ESM-1-2-HAM | 1.9^o^ × 1.9^o^ | r1i1p1f1 | HAMMOZ-Consortium / Switzerland, Germany, UK, Finland | Neubauer et al 2019 |
| MPI-ESM1-2-HR | 0.9^o^ × 0.9^o^ | r1i1p1f1 | Max Planck Institute for Meteorology (MPI-M) / Germany | Müller et al 2018 |
| MPI-ESM1-2-LR | 1.9^o^ × 1.9^o^ | r1i1p1f1 |  | Mauritsen et al 2019 |
| MRI-ESM2-0 | 1.1^o^ × 1.1^o^ | r1i1p1f1 | Meteorological Research Institute (MRI) / Japan | Yukimoto et al 2019 |
| NESM3 | 1.9^o^ × 1.9^o^ | r1i1p1f1 | Nanjing University of Information Science and Technology (NUIST) / China | Cao et al 2018 |
| NorCPM1 | 2.0^o^ × 2.5^o^ | r1i1p1f1 | NorESM Climate modeling Consortium (NCC) / Norway | Bethke et al 2021 |
| NorESM2-LM | 2.0^o^ × 2.5^o^ | r1i1p1f1 |  | Seland et al 2020 |
| NorESM2-MM | 0.9^o^ × 1.3^o^ | r1i1p1f1 |  |  |
| SAM0-UNICON | 0.9^o^ × 1.3^o^ | r1i1p1f1 | Seoul National University (SNU) / Rep. of Korea | Park et al 2019 |
| TaiESM1 | 0.9^o^ × 1.3^o^ | r1i1p1f1 | Research Center for Environmental Changes, Academia Sinica (AS-RCEC) / China | W.-L. Lee et al 2020 |
| UKESM1-0-LL | 1.3^o^ × 1.9^o^ | r1i1p1f2 | Natural Environment Research Council; Met Office Hadley Centre (NERC-MOHC) / UK | Sellar et al 2019 |

**Supplementary Table S2.** Scores of top-10 models, (52) ensemble, and (T10) ensemble for Holdridge’s Life Zones.

| **Model** | **Climate Cats.**  **(κ)** | **Precipitation**  **(R^2^)** | **Temperature**  **(R^2^)** |
| --- | --- | --- | --- |
| CESM2 | 0.64 | 0.77 | 0.98 |
| EC-Earth3 | 0.64 | 0.73 | 0.96 |
| EC-Earth3-Veg | 0.66 | 0.75 | 0.97 |
| EC-Earth3-Veg-LR | 0.64 | 0.75 | 0.96 |
| HadGEM3-GC31-LL | 0.65 | 0.75 | 0.98 |
| HadGEM3-GC31-MM | 0.69 | 0.76 | 0.98 |
| MRI-ESM2-0 | 0.66 | 0.72 | 0.98 |
| NorESM2-MM | 0.64 | 0.80 | 0.97 |
| TaiESM1 | 0.63 | 0.68 | 0.97 |
| UKESM1-0-LL | 0.66 | 0.75 | 0.97 |
| Ensemble (52 models) | 0.69 | 0.79 | 0.98 |
| Top-10 ensemble | **0.69** | **0.81** | **0.98** |

**Supplementary Table S3.** Scores of top-10 models, (52) ensemble, and (T10) ensemble for Köppen climate.

| **Model** | **Climate Cats.**  **(κ)** | **Precipitation**  **(R^2^)** | **Temperature**  **(R^2^)** |
| --- | --- | --- | --- |
| ACCESS-ESM1-5 | 0.68 | 0.69 | 0.97 |
| CESM2 | 0.69 | 0.77 | 0.98 |
| CIESM | 0.69 | 0.74 | 0.97 |
| EC-Earth3-Veg-LR | 0.69 | 0.75 | 0.96 |
| GFDL-ESM4 | 0.71 | 0.74 | 0.97 |
| HadGEM3-GC31-LL | 0.72 | 0.75 | 0.98 |
| HadGEM3-GC31-MM | 0.76 | 0.76 | 0.98 |
| MRI-ESM2-0 | 0.71 | 0.72 | 0.98 |
| TaiESM1 | 0.69 | 0.68 | 0.97 |
| UKESM1-0-LL | 0.71 | 0.75 | 0.97 |
| Ensemble (52 models) | 0.74 | 0.79 | 0.98 |
| Top-10 ensemble | **0.76** | **0.81** | **0.98** |

**Supplementary Table S4.** Scores of top-10 models, (52) ensemble, and (T10) ensemble for revised Thornthwaite.

| **Model** | **Climate Cats.**  **(κ)** | **Precipitation**  **(R^2^)** | **Temperature**  **(R^2^)** |
| --- | --- | --- | --- |
| CESM2-WACCM | 0.34 | 0.77 | 0.98 |
| EC-Earth3 | 0.37 | 0.73 | 0.96 |
| EC-Earth3-Veg | 0.37 | 0.75 | 0.97 |
| EC-Earth3-Veg-LR | 0.36 | 0.75 | 0.96 |
| HadGEM3-GC31-LL | 0.35 | 0.75 | 0.98 |
| HadGEM3-GC31-MM | 0.39 | 0.76 | 0.98 |
| MRI-ESM2-0 | 0.34 | 0.72 | 0.98 |
| NorESM2-MM | 0.36 | 0.80 | 0.97 |
| TaiESM1 | 0.36 | 0.68 | 0.97 |
| UKESM1-0-LL | 0.35 | 0.75 | 0.97 |
| Ensemble (52 models) | 0.42 | 0.79 | 0.98 |
| Top-10 ensemble | **0.45** | **0.81** | **0.98** |

**Supplementary Table S5**. Precipitation scores (R^2^) of 52 CMIP6 models and ensemble mean for two reference datasets: CRU and GPCC.

| **Model** | **GPCC** | **CRU** |
| --- | --- | --- |
| ACCESS-CM2 | 0.74 | 0.72 |
| ACCESS-ESM1-5 | 0.72 | 0.69 |
| AWI-CM-1-1-MR | 0.67 | 0.65 |
| AWI-ESM-1-1-LR | 0.67 | 0.59 |
| BCC-CSM2-MR | 0.67 | 0.64 |
| BCC-ESM1 | 0.67 | 0.65 |
| CAMS-CSM1-0 | 0.67 | 0.55 |
| CanESM5-CanOE | 0.67 | 0.64 |
| CanESM5 | 0.63 | 0.64 |
| CAS-ESM2-0 | 0.45 | 0.45 |
| CESM2-FV2 | 0.72 | 0.71 |
| CESM2 | 0.78 | 0.77 |
| CESM2-WACCM-FV2 | 0.70 | 0.70 |
| CESM2-WACCM | 0.70 | 0.77 |
| CIESM | 0.74 | 0.74 |
| CMCC-CM2-SR5 | 0.66 | 0.62 |
| CNRM-CM6-1-HR | 0.57 | 0.58 |
| CNRM-CM6-1 | 0.58 | 0.60 |
| CNRM-ESM2-1 | 0.56 | 0.57 |
| E3SM-1-0 | 0.68 | 0.67 |
| E3SM-1-1-ECA | 0.70 | 0.67 |
| E3SM-1-1 | 0.71 | 0.69 |
| EC-Earth3 | 0.73 | 0.73 |
| EC-Earth3-Veg-LR | 0.77 | 0.75 |
| EC-Earth3-Veg | 0.77 | 0.75 |
| FGOALS-f3-L | 0.50 | 0.48 |
| FGOALS-g3 | 0.44 | 0.44 |
| FIO-ESM-2-0 | 0.69 | 0.67 |
| GFDL-ESM4 | 0.74 | 0.74 |
| GISS-E2-1-G-CC | 0.59 | 0.60 |
| GISS-E2-1-G | 0.58 | 0.60 |
| GISS-E2-1-H | 0.45 | 0.47 |
| HadGEM3-GC31-LL | 0.78 | 0.75 |
| HadGEM3-GC31-MM | 0.79 | 0.76 |
| INM-CM4-8 | 0.73 | 0.72 |
| INM-CM5-0 | 0.73 | 0.72 |
| IPSL-CM6A-LR | 0.61 | 0.62 |
| KACE-1-0-G | 0.74 | 0.74 |
| MCM-UA-1-0 | 0.65 | 0.64 |
| MIROC6 | 0.70 | 0.69 |
| MIROC-ES2L | 0.68 | 0.68 |
| MPI-ESM-1-2-HAM | 0.61 | 0.59 |
| MPI-ESM1-2-HR | 0.69 | 0.67 |
| MPI-ESM1-2-LR | 0.67 | 0.66 |
| MRI-ESM2-0 | 0.72 | 0.72 |
| NESM3 | 0.70 | 0.70 |
| NorCPM1 | 0.59 | 0.58 |
| NorESM2-LM | 0.74 | 0.76 |
| NorESM2-MM | 0.80 | 0.80 |
| SAM0-UNICON | 0.74 | 0.72 |
| TaiESM1 | 0.71 | 0.68 |
| UKESM1-0-LL | 0.76 | 0.75 |
| ENSEMBLE(52) | 0.80 | 0.79 |
| CRU | 0.92 | 1.00 |
| GPCC | 1.00 | 0.92 |

**References**

Andrews, M.B., Ridley, J.K., Wood, R.A., Andrews, T., Blockley, E.W., Booth, B., Burke, E., Dittus, A.J., Florek, P., Gray, L.J., Haddad, S., Hardiman, S.C., Hermanson, L., Hodson, D., Hogan, E., Jones, G.S., Knight, J.R., Kuhlbrodt, T., Misios, S., Mizielinski, M.S., Ringer, M.A., Robson, J., Sutton, R.T., 2020. Historical Simulations With HadGEM3-GC3.1 for CMIP6. Journal of Advances in Modeling Earth Systems 12, e2019MS001995. https://doi.org/10.1029/2019MS001995

Bao, Y., Song, Z., Qiao, F., 2020. FIO-ESM Version 2.0: Model Description and Evaluation. Journal of Geophysical Research: Oceans 125, e2019JC016036. https://doi.org/10.1029/2019JC016036

Bethke, I., Wang, Y., Counillon, F., Keenlyside, N., Kimmritz, M., Fransner, F., Samuelsen, A., Langehaug, H., Svendsen, L., Chiu, P.-G., Passos, L., Bentsen, M., Guo, C., Gupta, A., Tjiputra, J., Kirkevåg, A., Olivié, D., Seland, Ø., Solsvik Vågane, J., Fan, Y., Eldevik, T., 2021. NorCPM1 and its contribution to CMIP6 DCPP. Geoscientific Model Development 14, 7073–7116. https://doi.org/10.5194/gmd-14-7073-2021

Bi, D., Dix, M., Marsland, S., O’Farrell, S., Sullivan, A., Bodman, R., Law, R., Harman, I., Srbinovsky, J., Rashid, H.A., Dobrohotoff, P., Mackallah, C., Yan, H., Hirst, A., Savita, A., Dias, F.B., Woodhouse, M., Fiedler, R., Heerdegen, A., Bi, D., Dix, M., Marsland, S., O’Farrell, S., Sullivan, A., Bodman, R., Law, R., Harman, I., Srbinovsky, J., Rashid, H.A., Dobrohotoff, P., Mackallah, C., Yan, H., Hirst, A., Savita, A., Dias, F.B., Woodhouse, M., Fiedler, R., Heerdegen, A., 2020. Configuration and spin-up of ACCESS-CM2, the new generation Australian Community Climate and Earth System Simulator Coupled Model. JSHESS 70, 225–251. https://doi.org/10.1071/ES19040

Boucher, O., Servonnat, J., Albright, A.L., Aumont, O., Balkanski, Y., Bastrikov, V., Bekki, S., Bonnet, R., Bony, S., Bopp, L., Braconnot, P., Brockmann, P., Cadule, P., Caubel, A., Cheruy, F., Codron, F., Cozic, A., Cugnet, D., D’Andrea, F., Davini, P., Lavergne, C. de, Denvil, S., Deshayes, J., Devilliers, M., Ducharne, A., Dufresne, J.-L., Dupont, E., Éthé, C., Fairhead, L., Falletti, L., Flavoni, S., Foujols, M.-A., Gardoll, S., Gastineau, G., Ghattas, J., Grandpeix, J.-Y., Guenet, B., Guez, L., E., Guilyardi, E., Guimberteau, M., Hauglustaine, D., Hourdin, F., Idelkadi, A., Joussaume, S., Kageyama, M., Khodri, M., Krinner, G., Lebas, N., Levavasseur, G., Lévy, C., Li, L., Lott, F., Lurton, T., Luyssaert, S., Madec, G., Madeleine, J.-B., Maignan, F., Marchand, M., Marti, O., Mellul, L., Meurdesoif, Y., Mignot, J., Musat, I., Ottlé, C., Peylin, P., Planton, Y., Polcher, J., Rio, C., Rochetin, N., Rousset, C., Sepulchre, P., Sima, A., Swingedouw, D., Thiéblemont, R., Traore, A.K., Vancoppenolle, M., Vial, J., Vialard, J., Viovy, N., Vuichard, N., 2020. Presentation and Evaluation of the IPSL-CM6A-LR Climate Model. Journal of Advances in Modeling Earth Systems 12, e2019MS002010. https://doi.org/10.1029/2019MS002010

Cao, J., Wang, B., Yang, Y.-M., Ma, L., Li, J., Sun, B., Bao, Y., He, J., Zhou, X., Wu, L., 2018. The NUIST Earth System Model (NESM) version 3: Description and preliminary evaluation. Geoscientific Model Development 11, 2975–2993. https://doi.org/10.5194/gmd-11-2975-2018

Cherchi, A., Fogli, P.G., Lovato, T., Peano, D., Iovino, D., Gualdi, S., Masina, S., Scoccimarro, E., Materia, S., Bellucci, A., Navarra, A., 2019. Global Mean Climate and Main Patterns of Variability in the CMCC-CM2 Coupled Model. Journal of Advances in Modeling Earth Systems 11, 185–209. https://doi.org/10.1029/2018MS001369

Danabasoglu, G., Lamarque, J.-F., Bacmeister, J., Bailey, D.A., DuVivier, A.K., Edwards, J., Emmons, L.K., Fasullo, J., Garcia, R., Gettelman, A., Hannay, C., Holland, M.M., Large, W.G., Lauritzen, P.H., Lawrence, D.M., Lenaerts, J.T.M., Lindsay, K., Lipscomb, W.H., Mills, M.J., Neale, R., Oleson, K.W., Otto‐Bliesner, B., Phillips, A.S., Sacks, W., Tilmes, S., Kampenhout, L. van, Vertenstein, M., Bertini, A., Dennis, J., Deser, C., Fischer, C., Fox‐Kemper, B., Kay, J.E., Kinnison, D., Kushner, P.J., Larson, V.E., Long, M.C., Mickelson, S., Moore, J.K., Nienhouse, E., Polvani, L., Rasch, P.J., Strand, W.G., 2020. The Community Earth System Model Version 2 (CESM2). Journal of Advances in Modeling Earth Systems 12, e2019MS001916. https://doi.org/10.1029/2019MS001916

Delworth, T., Stouffer, R., Dixon, K., Spelman, M., Knutson, T., Broccoli, A., Kushner, P., Wetherald, R., 2002. Review of simulations of climate variability and change with the GFDL R30 coupled climate model. Climate Dynamics 19, 555–574. https://doi.org/10.1007/s00382-002-0249-5

Döscher, R., Acosta, M., Alessandri, A., Anthoni, P., Arsouze, T., Bergman, T., Bernardello, R., Boussetta, S., Caron, L.-P., Carver, G., Castrillo, M., Catalano, F., Cvijanovic, I., Davini, P., Dekker, E., Doblas-Reyes, F.J., Docquier, D., Echevarria, P., Fladrich, U., Fuentes-Franco, R., Gröger, M., v. Hardenberg, J., Hieronymus, J., Karami, M.P., Keskinen, J.-P., Koenigk, T., Makkonen, R., Massonnet, F., Ménégoz, M., Miller, P.A., Moreno-Chamarro, E., Nieradzik, L., van Noije, T., Nolan, P., O’Donnell, D., Ollinaho, P., van den Oord, G., Ortega, P., Prims, O.T., Ramos, A., Reerink, T., Rousset, C., Ruprich-Robert, Y., Le Sager, P., Schmith, T., Schrödner, R., Serva, F., Sicardi, V., Sloth Madsen, M., Smith, B., Tian, T., Tourigny, E., Uotila, P., Vancoppenolle, M., Wang, S., Wårlind, D., Willén, U., Wyser, K., Yang, S., Yepes-Arbós, X., Zhang, Q., 2022. The EC-Earth3 Earth system model for the Coupled Model Intercomparison Project 6. Geoscientific Model Development 15, 2973–3020. https://doi.org/10.5194/gmd-15-2973-2022

Dunne, J.P., Horowitz, L.W., Adcroft, A.J., Ginoux, P., Held, I.M., John, J.G., Krasting, J.P., Malyshev, S., Naik, V., Paulot, F., Shevliakova, E., Stock, C.A., Zadeh, N., Balaji, V., Blanton, C., Dunne, K.A., Dupuis, C., Durachta, J., Dussin, R., Gauthier, P.P.G., Griffies, S.M., Guo, H., Hallberg, R.W., Harrison, M., He, J., Hurlin, W., McHugh, C., Menzel, R., Milly, P.C.D., Nikonov, S., Paynter, D.J., Ploshay, J., Radhakrishnan, A., Rand, K., Reichl, B.G., Robinson, T., Schwarzkopf, D.M., Sentman, L.T., Underwood, S., Vahlenkamp, H., Winton, M., Wittenberg, A.T., Wyman, B., Zeng, Y., Zhao, M., 2020. The GFDL Earth System Model Version 4.1 (GFDL-ESM 4.1): Overall Coupled Model Description and Simulation Characteristics. Journal of Advances in Modeling Earth Systems 12, e2019MS002015. https://doi.org/10.1029/2019MS002015

Golaz, J.-C., Caldwell, P.M., Roekel, L.P.V., Petersen, M.R., Tang, Q., Wolfe, J.D., Abeshu, G., Anantharaj, V., Asay‐Davis, X.S., Bader, D.C., Baldwin, S.A., Bisht, G., Bogenschutz, P.A., Branstetter, M., Brunke, M.A., Brus, S.R., Burrows, S.M., Cameron‐Smith, P.J., Donahue, A.S., Deakin, M., Easter, R.C., Evans, K.J., Feng, Y., Flanner, M., Foucar, J.G., Fyke, J.G., Griffin, B.M., Hannay, C., Harrop, B.E., Hoffman, M.J., Hunke, E.C., Jacob, R.L., Jacobsen, D.W., Jeffery, N., Jones, P.W., Keen, N.D., Klein, S.A., Larson, V.E., Leung, L.R., Li, H.-Y., Lin, W., Lipscomb, W.H., Ma, P.-L., Mahajan, S., Maltrud, M.E., Mametjanov, A., McClean, J.L., McCoy, R.B., Neale, R.B., Price, S.F., Qian, Y., Rasch, P.J., Eyre, J.E.J.R., Riley, W.J., Ringler, T.D., Roberts, A.F., Roesler, E.L., Salinger, A.G., Shaheen, Z., Shi, X., Singh, B., Tang, J., Taylor, M.A., Thornton, P.E., Turner, A.K., Veneziani, M., Wan, H., Wang, H., Wang, S., Williams, D.N., Wolfram, P.J., Worley, P.H., Xie, S., Yang, Y., Yoon, J.-H., Zelinka, M.D., Zender, C.S., Zeng, X., Zhang, C., Zhang, K., Zhang, Y., Zheng, X., Zhou, T., Zhu, Q., 2019. The DOE E3SM Coupled Model Version 1: Overview and Evaluation at Standard Resolution. Journal of Advances in Modeling Earth Systems 11, 2089–2129. https://doi.org/10.1029/2018MS001603

Hajima, T., Watanabe, M., Yamamoto, A., Tatebe, H., Noguchi, M.A., Abe, M., Ohgaito, R., Ito, Akinori, Yamazaki, D., Okajima, H., Ito, Akihiko, Takata, K., Ogochi, K., Watanabe, S., Kawamiya, M., 2020. Development of the MIROC-ES2L Earth system model and the evaluation of biogeochemical processes and feedbacks. Geoscientific Model Development 13, 2197–2244. https://doi.org/10.5194/gmd-13-2197-2020

He, B., Bao, Q., Wang, X., Zhou, L., Wu, X., Liu, Y., Wu, G., Chen, K., He, S., Hu, W., Li, Jiandong, Li, Jinxiao, Nian, G., Wang, L., Yang, J., Zhang, M., Zhang, X., 2019. CAS FGOALS-f3-L Model Datasets for CMIP6 Historical Atmospheric Model Intercomparison Project Simulation. Adv. Atmos. Sci. 36, 771–778. https://doi.org/10.1007/s00376-019-9027-8

Kelley, M., Schmidt, G.A., Nazarenko, L.S., Bauer, S.E., Ruedy, R., Russell, G.L., Ackerman, A.S., Aleinov, I., Bauer, M., Bleck, R., Canuto, V., Cesana, G., Cheng, Y., Clune, T.L., Cook, B.I., Cruz, C.A., Genio, A.D.D., Elsaesser, G.S., Faluvegi, G., Kiang, N.Y., Kim, D., Lacis, A.A., Leboissetier, A., LeGrande, A.N., Lo, K.K., Marshall, J., Matthews, E.E., McDermid, S., Mezuman, K., Miller, R.L., Murray, L.T., Oinas, V., Orbe, C., García‐Pando, C.P., Perlwitz, J.P., Puma, M.J., Rind, D., Romanou, A., Shindell, D.T., Sun, S., Tausnev, N., Tsigaridis, K., Tselioudis, G., Weng, E., Wu, J., Yao, M.-S., 2020. GISS-E2.1: Configurations and Climatology. Journal of Advances in Modeling Earth Systems 12, e2019MS002025. https://doi.org/10.1029/2019MS002025

Kuhlbrodt, T., Jones, C.G., Sellar, A., Storkey, D., Blockley, E., Stringer, M., Hill, R., Graham, T., Ridley, J., Blaker, A., Calvert, D., Copsey, D., Ellis, R., Hewitt, H., Hyder, P., Ineson, S., Mulcahy, J., Siahaan, A., Walton, J., 2018. The Low-Resolution Version of HadGEM3 GC3.1: Development and Evaluation for Global Climate. Journal of Advances in Modeling Earth Systems 10, 2865–2888. https://doi.org/10.1029/2018MS001370

Lee, J., Kim, Jisun, Sun, M.-A., Kim, B.-H., Moon, H., Sung, H.M., Kim, Jinwon, Byun, Y.-H., 2020. Evaluation of the Korea Meteorological Administration Advanced Community Earth-System model (K-ACE). Asia-Pacific J Atmos Sci 56, 381–395. https://doi.org/10.1007/s13143-019-00144-7

Lee, W.L., Wang, Y.C., Shiu, C.J., Tsai, I.C., Tu, C.Y., Lan, Y.Y., Chen, J.P., Pan, H.L., Hsu, H.H., 2020. Taiwan Earth System Model Version 1: description and evaluation of mean state. Geoscientific Model Development 13, 3887–3904. https://doi.org/10.5194/gmd-13-3887-2020

Li, L., Yu, Y., Tang, Y., Lin, P., Xie, J., Song, M., Dong, L., Zhou, T., Liu, L., Wang, Lu, Pu, Y., Chen, X., Chen, L., Xie, Z., Liu, Hongbo, Zhang, L., Huang, X., Feng, T., Zheng, W., Xia, K., Liu, Hailong, Liu, J., Wang, Y., Wang, Longhuan, Jia, B., Xie, F., Wang, B., Zhao, S., Yu, Z., Zhao, B., Wei, J., 2020. The Flexible Global Ocean-Atmosphere-Land System Model Grid-Point Version 3 (FGOALS-g3): Description and Evaluation. Journal of Advances in Modeling Earth Systems 12, e2019MS002012. https://doi.org/10.1029/2019MS002012

Lin, Y., Huang, X., Liang, Y., Qin, Y., Xu, S., Huang, W., Xu, F., Liu, L., Wang, Y., Peng, Y., Wang, L., Xue, W., Fu, H., Zhang, G.J., Wang, B., Li, R., Zhang, C., Lu, H., Yang, K., Luo, Yong, Bai, Y., Song, Z., Wang, M., Zhao, W., Zhang, F., Xu, J., Zhao, X., Lu, C., Chen, Y., Luo, Yiqi, Hu, Y., Tang, Q., Chen, D., Yang, G., Gong, P., 2020. Community Integrated Earth System Model (CIESM): Description and Evaluation. Journal of Advances in Modeling Earth Systems 12, e2019MS002036. https://doi.org/10.1029/2019MS002036

Mauritsen, T., Bader, J., Becker, T., Behrens, J., Bittner, M., Brokopf, R., Brovkin, V., Claussen, M., Crueger, T., Esch, M., Fast, I., Fiedler, S., Fläschner, D., Gayler, V., Giorgetta, M., Goll, D.S., Haak, H., Hagemann, S., Hedemann, C., Hohenegger, C., Ilyina, T., Jahns, T., Jimenéz‐de‐la‐Cuesta, D., Jungclaus, J., Kleinen, T., Kloster, S., Kracher, D., Kinne, S., Kleberg, D., Lasslop, G., Kornblueh, L., Marotzke, J., Matei, D., Meraner, K., Mikolajewicz, U., Modali, K., Möbis, B., Müller, W.A., Nabel, J.E.M.S., Nam, C.C.W., Notz, D., Nyawira, S.-S., Paulsen, H., Peters, K., Pincus, R., Pohlmann, H., Pongratz, J., Popp, M., Raddatz, T.J., Rast, S., Redler, R., Reick, C.H., Rohrschneider, T., Schemann, V., Schmidt, H., Schnur, R., Schulzweida, U., Six, K.D., Stein, L., Stemmler, I., Stevens, B., Storch, J.-S. von, Tian, F., Voigt, A., Vrese, P., Wieners, K.-H., Wilkenskjeld, S., Winkler, A., Roeckner, E., 2019. Developments in the MPI-M Earth System Model version 1.2 (MPI-ESM1.2) and Its Response to Increasing CO2. Journal of Advances in Modeling Earth Systems 11, 998–1038. https://doi.org/10.1029/2018MS001400

Müller, W.A., Jungclaus, J.H., Mauritsen, T., Baehr, J., Bittner, M., Budich, R., Bunzel, F., Esch, M., Ghosh, R., Haak, H., Ilyina, T., Kleine, T., Kornblueh, L., Li, H., Modali, K., Notz, D., Pohlmann, H., Roeckner, E., Stemmler, I., Tian, F., Marotzke, J., 2018. A Higher-resolution Version of the Max Planck Institute Earth System Model (MPI-ESM1.2-HR). Journal of Advances in Modeling Earth Systems 10, 1383–1413. https://doi.org/10.1029/2017MS001217

Neubauer, D., Ferrachat, S., Siegenthaler-Le Drian, C., Stier, P., Partridge, D.G., Tegen, I., Bey, I., Stanelle, T., Kokkola, H., Lohmann, U., 2019. The global aerosol–climate model ECHAM6.3–HAM2.3 – Part 2: Cloud evaluation, aerosol radiative forcing, and climate sensitivity. Geoscientific Model Development 12, 3609–3639. https://doi.org/10.5194/gmd-12-3609-2019

Park, S., Shin, J., Kim, S., Oh, E., Kim, Y., 2019. Global Climate Simulated by the Seoul National University Atmosphere Model Version 0 with a Unified Convection Scheme (SAM0-UNICON). J. Climate 32, 2917–2949. https://doi.org/10.1175/JCLI-D-18-0796.1

Rong X.Y., Chen H.M., Xin Y.F., Su J.Z., Hua L.J., Zhang Z.Q., 2019. Introduction of CAMS-CSM model and its participation in CMIP6. Climate Change Research 15, 540–544. https://doi.org/10.12006/j.issn.1673-1719.2019.186

Séférian, R., Nabat, P., Michou, M., Saint‐Martin, D., Voldoire, A., Colin, J., Decharme, B., Delire, C., Berthet, S., Chevallier, M., Sénési, S., Franchisteguy, L., Vial, J., Mallet, M., Joetzjer, E., Geoffroy, O., Guérémy, J.-F., Moine, M.-P., Msadek, R., Ribes, A., Rocher, M., Roehrig, R., Salas‐y‐Mélia, D., Sanchez, E., Terray, L., Valcke, S., Waldman, R., Aumont, O., Bopp, L., Deshayes, J., Éthé, C., Madec, G., 2019. Evaluation of CNRM Earth System Model, CNRM-ESM2-1: Role of Earth System Processes in Present-Day and Future Climate. Journal of Advances in Modeling Earth Systems 11, 4182–4227. https://doi.org/10.1029/2019MS001791

Seland, Ø., Bentsen, M., Olivié, D., Toniazzo, T., Gjermundsen, A., Graff, L.S., Debernard, J.B., Gupta, A.K., He, Y.-C., Kirkevåg, A., Schwinger, J., Tjiputra, J., Aas, K.S., Bethke, I., Fan, Y., Griesfeller, J., Grini, A., Guo, C., Ilicak, M., Karset, I.H.H., Landgren, O., Liakka, J., Moseid, K.O., Nummelin, A., Spensberger, C., Tang, H., Zhang, Z., Heinze, C., Iversen, T., Schulz, M., 2020. Overview of the Norwegian Earth System Model (NorESM2) and key climate response of CMIP6 DECK, historical, and scenario simulations. Geoscientific Model Development 13, 6165–6200. https://doi.org/10.5194/gmd-13-6165-2020

Sellar, A.A., Jones, C.G., Mulcahy, J.P., Tang, Y., Yool, A., Wiltshire, A., O’Connor, F.M., Stringer, M., Hill, R., Palmieri, J., Woodward, S., Mora, L. de, Kuhlbrodt, T., Rumbold, S.T., Kelley, D.I., Ellis, R., Johnson, C.E., Walton, J., Abraham, N.L., Andrews, M.B., Andrews, T., Archibald, A.T., Berthou, S., Burke, E., Blockley, E., Carslaw, K., Dalvi, M., Edwards, J., Folberth, G.A., Gedney, N., Griffiths, P.T., Harper, A.B., Hendry, M.A., Hewitt, A.J., Johnson, B., Jones, A., Jones, C.D., Keeble, J., Liddicoat, S., Morgenstern, O., Parker, R.J., Predoi, V., Robertson, E., Siahaan, A., Smith, R.S., Swaminathan, R., Woodhouse, M.T., Zeng, G., Zerroukat, M., 2019. UKESM1: Description and Evaluation of the U.K. Earth System Model. Journal of Advances in Modeling Earth Systems 11, 4513–4558. https://doi.org/10.1029/2019MS001739

Semmler, T., Danilov, S., Gierz, P., Goessling, H.F., Hegewald, J., Hinrichs, C., Koldunov, N., Khosravi, N., Mu, L., Rackow, T., Sein, D.V., Sidorenko, D., Wang, Q., Jung, T., 2020. Simulations for CMIP6 With the AWI Climate Model AWI-CM-1-1. Journal of Advances in Modeling Earth Systems 12, e2019MS002009. https://doi.org/10.1029/2019MS002009

Swart, N.C., Cole, J.N.S., Kharin, V.V., Lazare, M., Scinocca, J.F., Gillett, N.P., Anstey, J., Arora, V., Christian, J.R., Hanna, S., Jiao, Y., Lee, W.G., Majaess, F., Saenko, O.A., Seiler, C., Seinen, C., Shao, A., Sigmond, M., Solheim, L., von Salzen, K., Yang, D., Winter, B., 2019. The Canadian Earth System Model version 5 (CanESM5.0.3). Geoscientific Model Development 12, 4823–4873. https://doi.org/10.5194/gmd-12-4823-2019

Tatebe, H., Ogura, T., Nitta, T., Komuro, Y., Ogochi, K., Takemura, T., Sudo, K., Sekiguchi, M., Abe, M., Saito, F., Chikira, M., Watanabe, S., Mori, M., Hirota, N., Kawatani, Y., Mochizuki, T., Yoshimura, K., Takata, K., O’ishi, R., Yamazaki, D., Suzuki, T., Kurogi, M., Kataoka, T., Watanabe, M., Kimoto, M., 2019. Description and basic evaluation of simulated mean state, internal variability, and climate sensitivity in MIROC6. Geoscientific Model Development 12, 2727–2765. https://doi.org/10.5194/gmd-12-2727-2019

Voldoire, A., Saint‐Martin, D., Sénési, S., Decharme, B., Alias, A., Chevallier, M., Colin, J., Guérémy, J.-F., Michou, M., Moine, M.-P., Nabat, P., Roehrig, R., Mélia, D.S. y, Séférian, R., Valcke, S., Beau, I., Belamari, S., Berthet, S., Cassou, C., Cattiaux, J., Deshayes, J., Douville, H., Ethé, C., Franchistéguy, L., Geoffroy, O., Lévy, C., Madec, G., Meurdesoif, Y., Msadek, R., Ribes, A., Sanchez‐Gomez, E., Terray, L., Waldman, R., 2019. Evaluation of CMIP6 DECK Experiments With CNRM-CM6-1. Journal of Advances in Modeling Earth Systems 11, 2177–2213. https://doi.org/10.1029/2019MS001683

Volodin, E., Gritsun, A., 2018. Simulation of observed climate changes in 1850–2014 with climate model INM-CM5. Earth System Dynamics 9, 1235–1242. https://doi.org/10.5194/esd-9-1235-2018

Volodin, E., Mortikov, E., Kostrykin, S., Galin, V., Lykossov, V.N., Gritsun, A., Diansky, N., Gusev, A., Iakovlev, N., Shestakova, A., Emelina, S.V., 2018. Simulation of the modern climate using the INM-CM48 climate model. https://doi.org/10.1515/rnam-2018-0032

Wu, T., Lu, Y., Fang, Y., Xin, X., Li, L., Li, W., Jie, W., Zhang, J., Liu, Y., Zhang, L., Zhang, F., Zhang, Yanwu, Wu, F., Li, J., Chu, M., Wang, Z., Shi, X., Liu, Xiangwen, Wei, M., Huang, A., Zhang, Yaocun, Liu, Xiaohong, 2019. The Beijing Climate Center Climate System Model (BCC-CSM): the main progress from CMIP5 to CMIP6. Geoscientific Model Development 12, 1573–1600. https://doi.org/10.5194/gmd-12-1573-2019

Wu, T., Zhang, F., Zhang, J., Jie, W., Zhang, Y., Wu, F., Li, L., Yan, J., Liu, X., Lu, X., Tan, H., Zhang, L., Wang, J., Hu, A., 2020. Beijing Climate Center Earth System Model version 1 (BCC-ESM1): model description and evaluation of aerosol simulations. Geoscientific Model Development 13, 977–1005. https://doi.org/10.5194/gmd-13-977-2020

Wyser, K., van Noije, T., Yang, S., von Hardenberg, J., O’Donnell, D., Döscher, R., 2020. On the increased climate sensitivity in the EC-Earth model from CMIP5 to CMIP6. Geoscientific Model Development 13, 3465–3474. https://doi.org/10.5194/gmd-13-3465-2020

Yukimoto, S., Kawai, H., Koshiro, T., Oshima, N., Yoshida, K., Urakawa, S., Tsujino, H., Deushi, M., Tanaka, T., Hosaka, M., Yabu, S., Yoshimura, H., Shindo, E., Mizuta, R., Obata, A., Adachi, Y., Ishii, M., 2019. The Meteorological Research Institute Earth System Model Version 2.0, MRI-ESM2.0: Description and Basic Evaluation of the Physical Component. Journal of the Meteorological Society of Japan. Ser. II 97, 931–965. https://doi.org/10.2151/jmsj.2019-051

Zhou, G., Zhang, Y., Jiang, J., Zhang, H., Wu, B., Cao, H., Wang, T., Hao, H., Zhu, J., Yuan, L., Zhang, M., 2020. Earth System Model: CAS-ESM. Frontiers of Data and Computing 2, 38–54. https://doi.org/10.11871/jfdc.issn.2096-742X.2020.01.004

Ziehn, T., Chamberlain, M.A., Law, R.M., Lenton, A., Bodman, R.W., Dix, M., Stevens, L., Wang, Y.-P., Srbinovsky, J., Ziehn, T., Chamberlain, M.A., Law, R.M., Lenton, A., Bodman, R.W., Dix, M., Stevens, L., Wang, Y.-P., Srbinovsky, J., 2020. The Australian Earth System Model: ACCESS-ESM1.5. JSHESS 70, 193–214. https://doi.org/10.1071/ES19035
